# Scaling Laws in Language Families

**DOI:** 10.3390/e27060588

**Published:** 2025-05-31

**Authors:** Maelyson Rolim Fonseca dos Santos, Marcelo Andrade de Filgueiras Gomes

**Affiliations:** 1 Instituto Federal de Educação, Ciência e Tecnologia de Pernambuco, Barreiros 55560-000, PE, Brazil; 2 Departamento de Física, Universidade Federal de Pernambuco, Recife 50670-901, PE, Brazil

**Keywords:** language families, scaling laws, Zipf distributions

## Abstract

This article investigates scaling laws within language families using data from over six thousand languages and analyzes emergent patterns observed in Zipf-like classification graphs. Both macroscopic (based on the number of languages by family) and microscopic (based on the number of speakers by language within a family) aspects of these classifications are examined. Particularly noteworthy is the discovery of a distinct division among the fourteen largest contemporary language families, excluding Afro-Asiatic and Nilo-Saharan languages. These families are found to be distributed across three language family quadruplets, each characterized by significantly different exponents in the Zipf graphs. This finding sheds light on the underlying structure and organization of major language families, revealing intriguing insights into the nature of linguistic diversity and distribution.

## 1. Introduction

Complex systems are extensively characterized by scaling symmetry and power law distributions. These phenomena are evident in a wide range of studies, such as those on cities [1], growth models [2], cellular automata [3], and cognitive sciences [4], among many others. Language also exhibits characteristics of complex systems [5,6,7] and currently several statistical laws related to linguistic studies are well established [8].

An intriguing aspect in the analysis of complex systems is the role of entropy as a measure of disorder and diversification. In the linguistic context, historical processes of migration, fragmentation, and interaction between populations could be analogously interpreted as an “entropic flow”, where ancestral languages diversify and spread across the Earth’s surface, akin to the entropy of mixing in fluids [9]. In thermodynamics, mixing entropy quantifies the increase in disorder when two gases or liquids irreversibly interpenetrate. Similarly, linguistic diversification might be viewed as a process that maximizes a “cultural entropy”, where the fragmentation of proto-languages into diverse families and subsequent contact and hybridization processes generate emergent statistical patterns [10,11]. The Zipf-like scaling laws observed in this study, with characteristic exponents for different family groupings, may reflect distinct stages of this entropic process. For instance, higher exponents could correspond to linguistically “more mixed” systems, where prolonged interactions and homogenizing demographic pressures reduce relative diversity, while lower exponents might indicate families in early stages of fragmentation, preserving more pronounced hierarchies. This entropic perspective provides a conceptual bridge between the historical dynamics of languages and physical formalisms of self-organization in non-equilibrium systems [12,13].

Notedly interesting on the interface between statistical studies and linguistics, Zipf’s law for written texts relates the word frequency to their corresponding rank and points to a robust power-law dependence between these variables with a scaling exponent close to unity [14]. In recent decades, the expansion of available corpora and computational power has facilitated the study of Zipf’s law across various language systems [15,16,17,18,19].

Additionally, other types of non-Zipfian scaling laws along several decades of variability were also found in the study of the distribution of living languages by Gomes et al. [20], as well as by Santos and Gomes [21]. Although these studies have explored the relations between linguistic diversity and certain geographical, demographic, and economic factors, they have not delved into the investigation of scaling within language families.

More than two decades ago, Zanette analyzed the distribution of language family sizes across seventeen families [22]. Wichmann later studied the distribution of family sizes using data from the fourteenth edition of Ethnologue [23], while Hammarstrom conducted a similar study based on his own language family classification from the sixteenth edition of Ethnologue [24]. In this paper, we first examine the distribution of language family sizes using a more recent dataset. Differently from all previous studies, we then present an original analysis of the distribution of language sizes within each family, focusing on the fourteen largest language families.

The structure of this paper is as follows: in Section 2 we discuss the materials and methods. Section 3 introduces a scaling law emerging from the classification of language families according to the number of languages. Moreover, in Section 3 we study and we present the distribution of language sizes as measured by the number of speakers of 14 largest contemporary language families and discuss the appearance of quadruplets of language families. Section 4 ends with a brief summary of our conclusions.

## 2. Materials and Methods

We analyzed data from the digital twentieth edition of Ethnologue [25], one of the most comprehensive and longstanding catalogs of the world’s languages, first published in 1951. Over the past decades, Ethnologue has been widely used in linguistic and interdisciplinary research. Although our analysis is based on a more recent edition, a critical review of earlier versions of this database was conducted by Hammarström [26]. This edition classifies 6711 living languages into 141 families. The data for this study were manually extracted from the online version of Ethnologue; each language entry was individually accessed and recorded to ensure fidelity to the original classification and demographic information. In our analysis presented in this section, similar to that carried out by Wichmann [23], we excluded 388 languages categorized as constructed languages, creoles, sign languages, language isolates, mixed languages, pidgins, and unclassified languages. These languages were excluded because they do not consistently follow historical or genealogical patterns that define language families, making their inclusion problematic for comparative statistical analysis. For instance, constructed and pidgin languages are often the result of artificial or contact-driven processes rather than common ancestry, while isolates and unclassified languages lack sufficient data to be confidently grouped into families. Their inclusion could distort the scaling relations we aim to investigate.

An important feature to highlight is the wide range in the number of languages per family, varying from a single language, as in the Carajá family, to over a thousand, as in the Niger–Congo family. Regarding this variation in family sizes, Greenhill proposed five possible explanations: family age, population size, technology (agriculture/language dispersal hypothesis), geography and ecology, and social factors [27].

We began by ranking the language families in decreasing order of size, following an approach similar to Zipf’s ranking of words in *Human Behavior and the Principle of Least Effort* [14]. Thus, we assign rank r=1 to the Niger–Congo family, composed of 1526 languages; rank r=2 to the Austronesian family, with 1224 languages; rank r=3 to the Trans-New Guinea family, with 478 languages; and so on. In this way, we can express the cardinal size—that is, the number of languages—$N_{F}$ in a family of rank *r* as

(1) $$N_{F}∼r^{-\theta }.$$

Observing that only ten families have more than one hundred languages, it is worth asking whether a similar pattern is also found within each family. If so, this would imply that, when classifying individual languages by their number of speakers *N*, we should observe

(2) $$N∼r^{-\kappa },$$

where *r* is the language rank and κ is the corresponding exponent. In the next section, we present the results obtained from this classification.

## 3. Results and Discussion

### 3.1. Macroscopic Aspects

**Figure 1:**
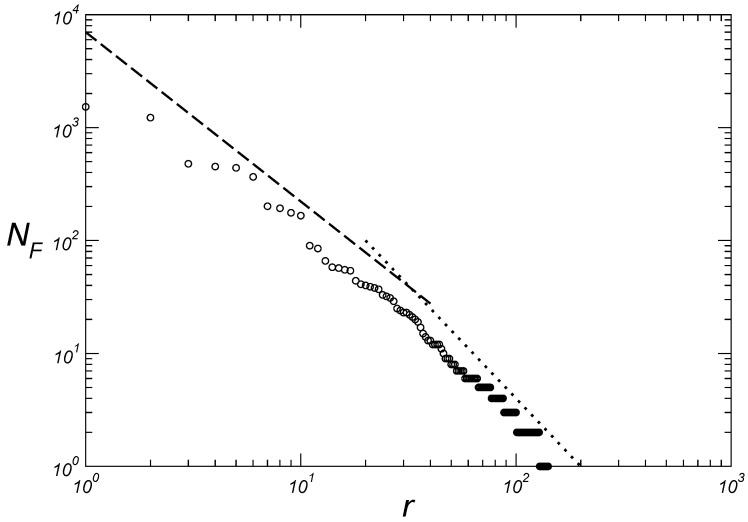
Number of languages $N_{F}$ of each language family as a function of their rank *r*. The dotted (dashed) line with slope −2.0 (−1.5) corresponds to scaling behavior associated with stable distributions [29]. The scaling exponents describe the data along approximately one decade of variability in the values of *r*.

The resulting graph of ranking the language families in decreasing order of size is shown in Figure 1. The choice of double logarithmic axes in this figure is justified so that the plotted points can be visualized linearly [28].

In Figure 1 can be viewed two different scaling behaviors associated with two distinct values for the exponent theta (Equation (1)): a first region with θ=1.5 for intermediary values of rank and a second region with θ=2.0 for large values of rank. The difference in exponents shown in Figure 1 may reflect the fact that larger families tend to be older and/or have more complex demographic histories. Importantly, the dotted and dashed lines in Figure 1 (and subsequent figures) do not represent statistical fits to the data. Instead, these lines act as qualitative guides to highlight proposed scaling regimes that capture the main observed trends. Such visual representations are common in exploratory analyses of complex systems, where strict parametric fitting may not adequately capture crossover behaviors or data heterogeneity. The distribution of the size of language families that we report here resembles the so-called Hollow Curve [27], a characteristic right-skewed distribution commonly observed in biological taxa, where a few groups are very large while most are small.

For large values of *r*, the exponent θ=2.0 referred in Figure 1 is close to the value (θ=1.905) previously obtained from the fourteenth edition of *Ethnologue* [23] but greater than the value (θ=1.38) obtained by Hammarström from their own language family classification from the sixteenth edition of *Ethnologue* [24]. This last value, however, seems more akin to the slope 1.5 observed for the intermediary rank values in Figure 1. Our result contradicts Zanette [22] who, analyzing a set of seventeen families from *A Guide to the World’s Languages* by Ruhlen [30], proposed that the number of languages would decrease exponentially with the family rank.

Hammarström points out that since linguistic differentiation occurs mainly through human migration, the cardinal size of a family can be considered a measure of the diffusive spread of a family [24]. From this perspective, we can understand that only a small number of language families are spread over large areas of the Earth’s surface. At the same time, it is possible to understand that most families have a small geographical reach.

### 3.2. Microscopic Aspects

**Figure 2:**
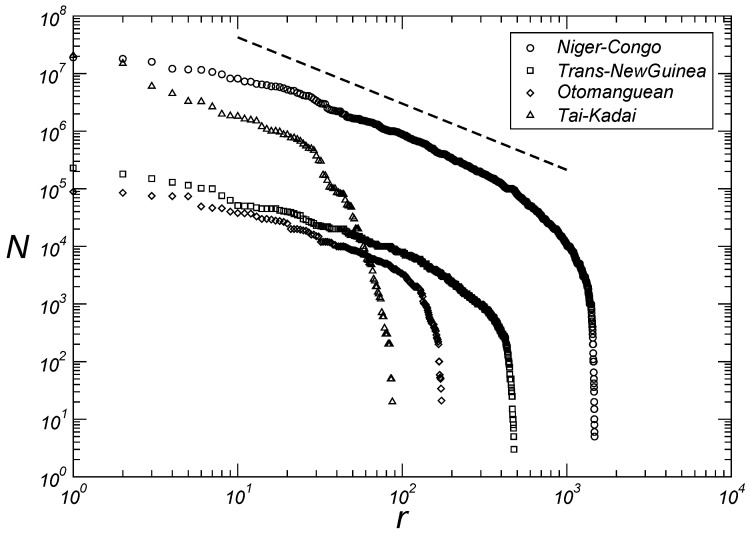
Number of speakers *N* by language as a function of rank *r* for the Niger–Congo, Trans-New Guinea, Otomanguean and Tai-Kadai families. The dashed line provides guidance for eye adjustments $N∼r^{-\kappa }$ with κ=1.15.

**Figure 3:**
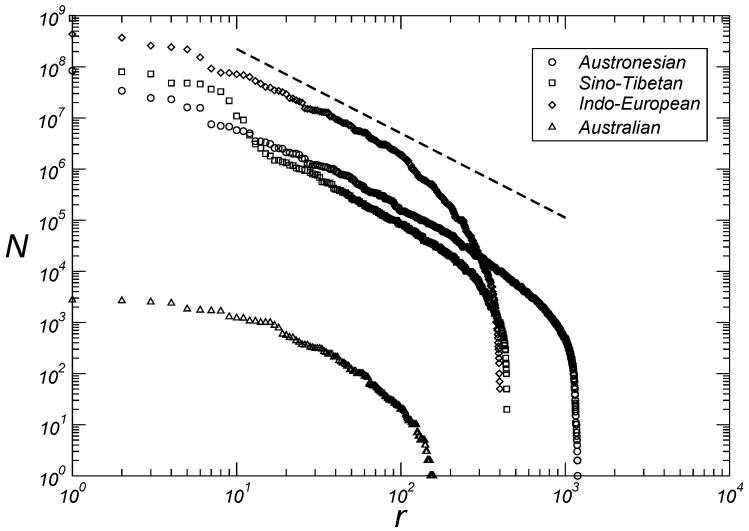
Number of speakers *N* by language as a function of rank *r* for the Austronesian, Sino-Tibetan, Indo-European, and Australian families. The dashed line provides guidance for eye adjustments $N∼r^{-\kappa }$ with κ=1.65.

**Figure 4:**
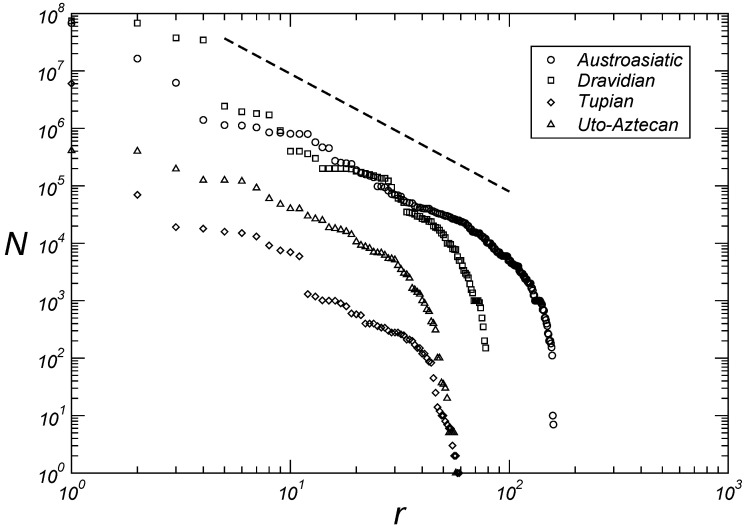
Number of speakers *N* by language as a function of rank *r* for the Austroasiatic, Dravidian, Tupian and Uto-Aztecan families. The dashed line provides guidance for eye adjustments $N∼r^{-\kappa }$ with κ=2.05.

Table 1 shows the kappa value for the fourteen largest language families according to the number of speakers (Equation (2)). In the following paragraphs we will discuss each of these families specifically and will show that, with the exception of the Afro-Asian and Nilo-Saharan families, the remaining twelve families are distributed in three quadruplets of language families grouped according to the exponent of Zipf’s distributions. At large rank values, the curves shown in Figure 2, Figure 3 and Figure 4 deviate from the classical Zipfian form, primarily due to the dominance of languages with very small speaker populations. This deviation likely arises from reduced viability or incomplete recording of languages with very few speakers.

The largest language family, the Niger–Congo, is composed of 1526 languages and encompasses nearly all native languages in Africa below the Sahara and is characterized by κ=1.2. Another family, consisting of over a thousand languages, is the Austronesian, with its 1224 languages scattered from Indonesia through the island of New Guinea to Easter Island. This family, originating from the region of Taiwan, has κ=1.6. New Guinea is also home to the Trans-New Guinea family, composed of 478 languages and characterized by κ=1.1. The Sino-Tibetan family has 452 languages but boasts over 380 times more speakers than the Trans-New Guinea family and it has κ=1.7. The Indo-European family includes almost all languages in Europe and many languages in the Asian continent. Among the top twenty languages globally, eleven belong to this family, totaling over three billion speakers distributed across 440 languages with κ=1.7.

The 366 languages that make up the Afro-Asiatic family likely descend from the language spoken by human groups that migrated from the African continent to the Middle East over 50,000 years ago. It was in Phoenician, a language of this family, that the first phonetic alphabet was constructed. Unlike the five largest families characterized by two exponent values (κ=1.15±0.05 and κ=1.65±0.05), the Afro-Asiatic family has κ=2.6, making this value the highest among the fourteen major language families. South of the Afro-Asiatic languages region lies another family whose κ value is also distinct from those two characteristic of the five largest families: comprising over 200 languages, the Nilo-Saharan family has κ=1.4.

The Australian family, although very diverse in the number of languages (193 in total), has experienced a significant decline in the number of speakers over the past centuries, making it the smallest family by this criterion among those discussed here. Australian languages with fewer than ten thousand speakers have κ=1.6, which is the same value reported for the Austronesian family. The Otomanguean family, characteristic of the current Mexican territory, also experienced a population reduction after colonization processes and presents κ=1.1. This value is identical to that reported for the Trans-New Guinea family.

The Austro-Asiatic language family, spoken from Southeast Asia to East India, ranks as the ninth-largest language family globally, with κ=2.0. Southeast Asia is also home to the Tai-Kadai family, boasting about half the number of languages as the Austro-Asiatic family, and it is characterized by κ=1.2. Moving to the Dravidian family, typical in South Asia, it boasts a linguistic population exceeding two hundred million speakers. Many of these languages persisted despite the expansion and dominance of Indo-European languages in the Indian region. The Dravidian family shares the same exponent as the Austro-Asiatic family, with κ=2.0.

The processes resulting from colonization irreversibly affected the distribution of languages on the American continent. Two of the language families most affected were the Tupian, in South America, and the Uto-Aztecan, in North America. These two families are characterized by κ=2.1. Together with the Austro-Asiatic and Dravidian families, these two families constitute a quadruplet characterized by κ=2.05±0.05.

Therefore, as discussed in the preceding paragraphs, with the exception of the Afro-Asiatic and Nilo-Saharan families, we have three quadruplets of language families grouped according to the exponent of the Zipf distributions. The first is composed of the Trans-New Guinea, Otomanguean, Niger–Congo, and Tai-Kadai families with κ=1.15±0.05, as seen in Figure 2. With κ=1.65±0.05, the second quadruplet comprises the families Austronesian, Australian, Sino-Tibetan, and Indo-European, as seen in Figure 3. And finally the third quadruplet with the families Austro-Asiatic, Dravidian, Tupian, and Uto-Aztecan has κ=2.05±0.05, as seen in Figure 4.

## 4. Conclusions

Here we present power laws emerging from the distributions of both the size of linguistic families according to the number of languages and the size of languages according to their respective number of speakers within each of the fourteen largest families, based on methods for classifying and ordering data from more than six thousand languages. In contrast to previous studies, we show in Figure 1 that the cardinal size of a language family is related to the rank of the family by two exponents (1.5 and 2.0). The first for intermediate values of rank and the second for large values of rank. We then show that for twelve language families (Niger–Congo, Trans-New Guinea, Otomanguean, Tai-Kadai; Austronesian, Sino-Tibetan, Indo-European, Australian; Austroasiatic, Dravidian, Tupian, Uto-Aztecan) out of the fourteen largest families, we have three quadruplets of language families grouped according to the exponent of the Zipf distributions, namely, κ=1.15±0.05 (Figure 2), κ=1.65±0.05 (Figure 3), and κ=2.05±0.05 (Figure 4). We believe that future research, with particular emphasis on detailed human migratory processes, should seek to understand (*i*) why these twelve language families form statistically well-characterized quadruplets according to the values of the exponents, and (*ii*) why the Afro-Asiatic and Nilo-Saharan families, both from the African continent, have different exponent values from those of the aforementioned quadruplets. Regarding this last aspect, it seems clear to us to expect that the continent where the greatest diversity of languages was originally generated should also present the greatest number of different emerging classes of statistical distributions that help to characterize this same diversity. Assessing whether these results hold across alternative datasets represents an important direction for future investigation.

## Figures and Tables

**Table 1:** Fourteen largest language families according to the number of languages $N_{F}$. The value *r* indicates the classification of the language according to this ordering and $r_{s}$ indicates the classification of the family according to the linguistic population *N*. The value κ is the exponent of the scaling law $N∼r^{-\kappa }$.

*r*	Family	$N_{F}$	*N* (in Millions)	$r_{s}$	κ
01	Niger–Congo	1526	458.90	03	1.2
02	Austronesian	1224	324.88	05	1.6
03	Trans-New Guinea	478	3.55	21	1.1
04	Sino-Tibetan	452	1355.71	02	1.7
05	Indo-European	440	3077.11	01	1.7
06	Afro-Asiatic	366	444.85	04	2.6
07	Nilo-Saharan	201	50.33	12	1.4
08	Australian	193	0.04	51	1.6
09	Otomanguean	176	1.68	24	1.1
10	Austro-Asiatic	166	104.99	09	2.0
11	Tai-Kadai	90	80.1	10	1.2
12	Dravidian	85	228.1	06	2.0
13	Tupian	66	6.2	19	2.1
14	Uto-Astecan	58	1.9	22	2.1

## Data Availability

No new data were created or analyzed in this study. The data used are publicly available through the online platform of the 20th edition of *Ethnologue* (https://www.ethnologue.com/20 (accessed on 14 July 2021)).
